# Increased vulnerability of rural children on antiretroviral therapy attending public health facilities in South Africa: a retrospective cohort study

**DOI:** 10.1186/1758-2652-13-46

**Published:** 2010-11-25

**Authors:** Geoffrey Fatti, Peter Bock, Ashraf Grimwood, Brian Eley

**Affiliations:** 1 Kheth'Impilo, Green Square, 37 Hares Crescent, Woodstock, 7925, Cape Town, South Africa; 2 Lung Clinical Research Unit, University of Cape Town Lung Institute, George Street, Mowbray, Cape Town, South Africa; 3 Primary Healthcare Directorate, University of Cape Town, Groote Schuur Hospital, Observatory, Cape Town, South Africa; 4 Paediatric Infectious Diseases Unit, Red Cross Children's Hospital, School of Child and Adolescent Health, University of Cape Town, Klipfontein Road, Rondebosch, Cape Town, South Africa

## Abstract

**Background:**

A large proportion of the 340,000 HIV-positive children in South Africa live in rural areas, yet there is little sub-Saharan data comparing rural paediatric antiretroviral therapy (ART) programme outcomes with urban facilities. We compared clinical, immunological and virological outcomes between children at seven rural and 37 urban facilities across four provinces in South Africa.

**Methods:**

We conducted a retrospective cohort study of routine data of children enrolled on ART between November 2003 and March 2008 in three settings, namely: urban residence and facility attendance (urban group); rural residence and facility attendance (rural group); and rural residents attending urban facilities (rural/urban group). Outcome measures were: death, loss to follow up (LTFU), virological suppression, and changes in CD4 percentage and weight-for-age-z (WAZ) scores. Kaplan-Meier estimates, logrank tests, multivariable Cox regression and generalized estimating equation models were used to compare outcomes between groups.

**Results:**

In total, 2332 ART-naïve children were included, (1727, 228 and 377 children in the urban, rural and rural/urban groups, respectively). At presentation, rural group children were older (6.7 vs. 5.6 and 5.8 years), had lower CD4 cell percentages (10.0% vs. 12.8% and 12.7%), lower WAZ scores (-2.06 vs. -1.46 and -1.41) and higher proportions with severe underweight (26% vs.15% and 15%) compared with the urban and rural/urban groups, respectively. Mortality was significantly higher in the rural group and LTFU significantly increased in the rural/urban group. After 24 months of ART, mortality probabilities were 3.4% (CI: 2.4-4.8%), 7.7% (CI: 4.5-13.0%) and 3.1% (CI: 1.7-5.6%) p = 0.0137; LTFU probabilities were 11.5% (CI: 9.3-14.0%), 8.8% (CI: 4.5-16.9%) and 16.6% (CI: 12.4-22.6%), p = 0.0028 in the urban, rural and rural/urban groups, respectively. The rural group had an increased adjusted mortality probability, adjusted hazards ratio 2.41 (CI: 1.25-4.67) and the rural/urban group had an increased adjusted LTFU probability, aHR 2.85 (CI: 1.41-5.79). The rural/urban group had a decreased adjusted probability of virological suppression compared with the urban group at any timepoint on treatment, adjusted odds ratio 0.67 (CI: 0.48-0.93).

**Conclusions:**

Rural HIV-positive children are a vulnerable group, exhibiting delayed access to ART and an increased risk of poor outcomes while on ART. Expansion of rural paediatric ART programmes, with future research exploring improvements to rural health system effectiveness, is required.

## Background

South Africa has the largest paediatric HIV epidemic and the largest paediatric antiretroviral treatment (ART) programme in the world [1]. By mid-2009, an estimated 340,000 children younger than 15 years of age were living with HIV infection [2], of whom approximately 70,000 were receiving ART [3]. Paediatric ART outcomes in South Africa have shown favourable short-term responses [4-7], with a recent cohort study involving more than 6000 children demonstrating that ≥80% achieved virological suppression during the first three years of treatment [8]. Despite the rapid scale up of ART programmes, marked inequities remain in access to early infant diagnosis and ART provision between different areas of the country [9].

Limited information on the outcomes of children managed in rural ART programmes is available in South Africa. A cohort from a single rural sub-district in the province of KwaZulu-Natal demonstrated encouraging short-term ART outcomes [10]. However, a large proportion of children developed virological failure and major drug-resistant mutations on first-line ART in another rural cohort [11]. Most paediatric ART studies in South Africa have included only urban children [4-8], with no direct comparisons between outcomes in rural and urban children. More than 40% of South Africa's population, however, lives in rural areas [12,13].

The prevalence of HIV in children and adults is as high in rural as in urban areas [14], and certain rural districts have reported extremely high infection rates, particularly among women [15]. A recent comparison of ART outcomes between urban and rural children at three Zambian sites demonstrated rural children having higher levels of malnutrition at presentation and having higher mortality rates while on treatment [16]. Difficulties in providing rural ART in sub-Saharan Africa include a lack of health personnel, lack of diagnostic facilities, difficulty and expense transporting medication to clinics, and a lack of staff training in paediatric ART delivery [10,15,17].

In order to further examine the effectiveness of providing rural paediatric ART in low-income settings, the aim of this study was to compare clinical, immunological and virological outcomes between rural and urban children on ART in a large cohort from multiple public health facilities in four provinces of South Africa.

## Methods

### Study design, setting and participants

A retrospective cohort study of children enrolled on ART was conducted at 44 routine public healthcare facilities supported by Absolute Return for Kids South Africa (subsequently named Kheth'Impilo), a non-governmental organization (NGO) that supports the scale up of ART in public sector clinics in South Africa. Seven rural and 37 urban sites that had electronic data collection systems used for patient monitoring purposes were included in the study. Facilities were distributed across four provinces (Western Cape, KwaZulu-Natal, Eastern Cape and Mpumalanga), with 12 facilities being secondary level hospitals and 32 being primary healthcare clinics. All sites treated both adults and children.

All ART-naïve children (<16 years) enrolled on ART between 1 November 2003 and 31 December 2007 with documented date of birth, gender and date of starting ART, and who had initiated triple combination ART, were included in the analyses. Children were selected to start ART according to the national Department of Health guidelines [18]. Briefly, children with modified World Health Organization (WHO) clinical stage III or stage IV disease, or a low CD4 cell percentage irrespective of disease stage (<20% in children under 18 months of age, or <15% if over 18 months old), or recurrent or prolonged hospitalization were eligible for ART.

Additionally, children were required to have an identifiable adult caregiver who could administer the medication. First-line ART consisted of two nucleoside reverse transcriptase inhibitors (NRTIs) plus a non-nucleoside reverse transcriptase inhibitor (NNRTI) for those aged older than three years or a protease inhibitor (PI) for those younger than three years. PI-containing regimes were used for children who were exposed to perinatal nevirapine, and stavudine was used in preference to zidovudine when home refrigeration was available. CD4 cell count and percentage was measured at ART initiation and at six-monthly intervals, and viral load was monitored six monthly on treatment. Patients were followed up until 31 March 2008 or until the NGO exited from a site.

Children were categorized into three groups defined by their place of residence and ART facility attended, namely: urban residence and urban ART facility attended (urban group); rural residence and rural facility attended (rural group); and rural residents attending urban facilities (rural/urban group). The Global Rural-Urban Mapping Project population definitions were used to assign urban or rural categorization, in which settlements with a population of less than 5000 people are classified as rural [19]. Population data were derived from the 2001 South African population census [20].

### Outcomes and case definitions

Outcome measures were: death, loss to follow up (LTFU), virological suppression, and changes in CD4 cell percentage and weight-for-age-z-scores (WAZ). Follow-up time was censored at 24 months after starting ART. LTFU was defined as no patient visit for three months after the last scheduled appointment was missed and viral load suppression as a viral load <400 copies/ml. Children who miss appointments would initially be traced by telephone and in certain cases, dependent on community health worker availability and if prior consent was obtained, a community health worker or district tracing team would visit the client's house (tracing systems are, however, not standardized between sites). CD4 cell counts and viral load were measured by the National Health Laboratory Services using the Panleucogating method [21] and the Nuclisens HIV1 QT assay (bioMerieux, Marcy-Etiole, Rhône), respectively.

### Data collection

Individual-level patient data were collected prospectively for routine monitoring purposes by designated site-based data capturers at each patient visit using Microsoft Access databases, which were pooled on a quarterly basis to a central data warehouse using standard operating procedures. Continual data cleaning and quality control routines were implemented to enhance data validity. Missing data values were attempted to be retrieved by hand searching paper-based patient records at facilities. The study was approved by the University of Cape Town Research Ethics committee, reference number 368/2008.

### Statistical analysis

Children were categorized to age groups of <1 year, 1-2 years, 2-5 years, 6-10 years, and ≥11 years. Baseline characteristics between groups were compared using the ANOVA, Kruskal-Wallis, Pearson's χ^2 ^and Bonferroni tests, as appropriate. Kaplan-Meier curves were fitted to estimate mortality and LTFU from the programme. The logrank test was used to compare groups. Multivariable Cox proportional hazards regression was used to assess group effect associated with death and LTFU until 24 months of ART, adjusting for baseline demographic and clinical variables and accounting for heterogeneity between individual site cohorts.

For regression analyses, severe immunodeficiency was defined according to WHO criteria [22]: CD4 percentage <25% or CD4 count <1500 cells/mm^3 ^for children younger than 12 months; CD4 percentage <20% or CD4 count <750 cells/mm^3 ^for children between 12 and 35 months; CD4 percentage <15% or CD4 count <350 cells/mm^3 ^for children between 36 and 59 months; and CD4 percentage <15% or CD4 count <200 cells/mm^3 ^for children five years and older. Due to the significant proportion of missing values for baseline weight and WHO clinical stage, a composite variable was created and named severe clinical status, defined as a WAZ score of <-3 (severe underweight) or a WHO stage ≥3 [23].

The influence of the availability of immunologic and clinical status variables with mortality and LTFU were assessed by considering missing values as a third category to the initially binary variables. When comparing groups, the group with the lowest Kaplan-Meier estimates of each outcome was selected as the comparative group. Subgroup analyses were additionally performed using regression models to compare group effect by including only children with all baseline variables being available and substituting WHO clinical stage and WAZ-score variables instead of severe clinical status.

Multivariate generalized estimating-equation population-averaged models were used to analyze group effect on virological suppression, CD4 cell percentage increase and WAZ-score increase until 24 months on ART, adjusting for baseline clinical and demographic variables. Gender and age standardized z-scores for weight and height were calculated using the Centres for Disease Control 2000 growth reference standards [24]. All statistical analyses were performed using Stata version 9.2 (Stata Corporation, College Station, Texas, USA).

## Results

Database records for a total of 3358 children who started ART were screened for eligibility for the study. A total of 1026 were excluded for the following reasons: 572 were ART experienced; 352 commenced ART after 31 December 2007; and 102 had unavailable demographic data. Thus, 2332 ART-naïve children from seven rural and 37 urban facilities in four provinces were included in the analysis, with 605 children living in rural areas.

There were 1727 (74.1%), 228 (9.8%) and 377 (16.2%) children in the urban, rural and rural/urban groups, respectively (Table 1). Children starting ART in the rural group were older, with a median age of 6.7 years compared with 5.6 years and 5.8 years (p = 0.0001) in the urban and rural/urban groups, respectively. Rural group children had the lowest median baseline CD4 cell percentage (10.0%) compared with 12.8% and 12.8% (p = 0.0003; Kruskal-Wallis test) in the urban and rural/urban groups, respectively.

**Table 1:** Baseline characteristics of ART-naïve children beginning antiretroviral therapy

	All	Urban	Rural	Rural/Urban	P value	**Group differing**^**a**^
	**(n = 2332)**	**(n = 1727)**	**(n = 228)**	**(n = 377)**		

**Median age, y (IQR)**	5.8 (3.0-9.0)	5.6 (2.8-8.9)	6.7 (4.3-10.0)	5.8 (3.2-9.3)	0.0001	Rural

**Age group categories, n (%)**					0.001	Rural

<1 year	129 (5.5)	112 (6.5)	3 (1.3)	14 (3.7)		

1-2 yrs	246 (10.6)	188 (10.9)	17 (7.5)	41 (10.9)		

2-5 yrs	834 (35.8)	628 (36.4)	72 (31.6)	134 (35.5)		

6-10 yrs	814 (34.8)	573 (33.2)	94 (41.2)	145 (38.5)		

≥11 yrs	311 (13.3)	226 (13.1)	42 (18.4)	43 (11.4)		

**Female, n (%)**	1174 (50.3)	887 (51.3)	98 (42.0)	189 (50.1)	0.059	Rural

**WHO clinical stage, n (%), (n = 1836)**					0.059	Rural

I/II	615 (33.5)	468 (33.1)	64 (41.8)	83 (31.0)		

III/IV	1221 (66.5)	947 (66.9)	89 (58.2)	185 (69.0)		

**Weight-for-age z-score, mean (95% CI), (n = 1572**)	-1.51 (-1.59 to -1.44)	-1.46 (-1.54 to -1.38)	-2.06 (-2.30 to -1.82)	-1.41 (-1.60 to -1.22)	<0.0001	Rural

**Weight-for-age z-score < -3, n (%)**	253 (16.1)	177 (15.0)	42 (25.9)	34 (14.9)	<0.002	Rural

**Height-for-age z-score, mean (95% CI), (n = 359)**	-1.96 (-2.11 to -1.82)	-1.91 (-2.01 to -1.75)	-2.26 (-2.70 to -1.82)	-1.96 (-2.26 to -1.27)	0.271	

**Severe clinical status, n (%), (n = 2057)**	549 (26.7)	399 (25.9)	53 (27.0)	97 (30.3)	0.265	

**CD4 cell percentage; median (IQR), (n = 1425)**	12.2 (7.0-18.0)	12.8 (7.1-19.0)	10.0 (6.0-14.2)	12.8 (6.3-18.1)	0.0003	Rural

**Absolute CD4 cell count (cells/mm^3^); median (IQR), (n = 1665)**	271 (54-630)	301 (50-685)	212 (77-405)	206 (24-607)	0.0019	Urban

**Severe immunodeficiency, n (%), (n = 1772)**	1285 (72.5)	933 (71.5)	172 (79.6)	180 (72.0)	0.045	Rural

**TB treatment at ART initiation, n (%), (n = 2291)**	79 (3.5)	51 (3.0)	8 (3.5)	20 (5.3)	0.090	

**Initial ART regimen, n (%), (n = 2280)**						

NNRTI-based	1714 (75.2)^a^	1211 (71.9)	210 (93.8)	293 (79.0)	<0.0001	Rural

PI-based	556 (24.4)^a^	467 (27.7)	14 (6.3)	75 (20.2)	<0.0001	Rural

Including d4t	2147 (94.2)^b^	1555 (92.3)	224 (100)	368 (99.2)	<0.0001	Urban

Including ZDV	143 (6.3)^b^	137 (8.1)	0 (0)	6 (1.6)	<0.000	Urban

**Province, n (%)**					<0.0001	Urban

Western Cape	552 (23.7)	552 (31.9)	0 (0)	0 (0)		

Eastern Cape	102 (4.4)	100 (5.8)	2 (0.9)	0 (0)		

KwaZulu-Natal	1654 (70.9)	1065(61.7)	212 (93.0)	377 (100)		

Mpumalanga	24 (1.0)	10 (0.6)	14 (6.1)	0 (0)		

^a^Indicates group differing significantly from the other groups

^b^10 children received d4t and ZDV, and did not receive an NNRTI or a PI

WHO: World Health Organization

The rural group also displayed the highest proportion with severe immunodeficiency (79%; 95% CI: 73.6-84.8%; p = 0.043). This group, however, displayed a trend toward less advanced baseline WHO clinical stage disease (58.2% with stage ≥ III compared with 66.9% and 69.0% in the urban and rural/urban groups, respectively, χ^2 ^p = 0.059), although no overall differences were found between groups in the proportions of patients with severe clinical status (p = 0.265). Children in the rural group had the lowest mean baseline WAZ score, being -2.06 (95% CI: -2.30 to -1.82; p <0.0001) and the highest proportion with severe underweight (25.9%; 95% CI: 19.3-33.4%; p = 0.002).

Seventy-nine children (3.5%) were documented as having started ART while taking antituberculous therapy, with no difference between groups. The majority (75.2%) of children commenced NNRTI-based regimens; rural children had the lowest proportion starting PI-based regimens (6.3%; p < 0.0001), reflecting their older age and the different recommended initial regimens for older and younger children. Urban children had a higher proportion starting regimens containing zidovudine (8.1%; p < 0.0001) instead of stavudine.

**Figure 1:**
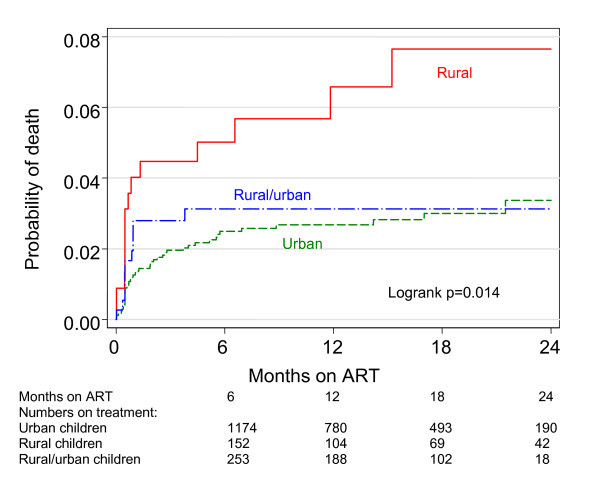
**Kaplan-Meier estimates of cumulative mortality between rural and urban children**.

Overall programme retention in care and mortality after 24 months of ART was 84.5% (95% CI: 82.2%-86.6%) and 3.8% (95% CI: 2.9%-4.9%), respectively. During the study period, 69 (3.0%) children died, with 44 (2.6%) children dying in the urban group, 14 (6.1%) in the rural group, and 11 (2.9%) in the rural/urban group (χ^2 ^p = 0.011). After 24 months of ART, mortality probabilities were significantly higher in the rural group, being 3.4% (95% CI: 2.4-4.8%), 7.7% (95% CI: 4.5-13.0%) and 3.1% (95% CI: 1.7-5.6%) in the urban, rural and rural/urban groups, respectively, logrank p = 0.0137 (Figure 1). Overall rates of mortality peaked during the first six months of treatment, with the highest rate in the rural group, being 5.4 (95% CI: 3.9-7.4), 11.4 (95% CI: 6.3-20.6) and 7.1 (95% CI: 3.9-12.8) deaths per 100 person-years in the urban, rural and rural/urban groups, repectively.

**Figure 2:**
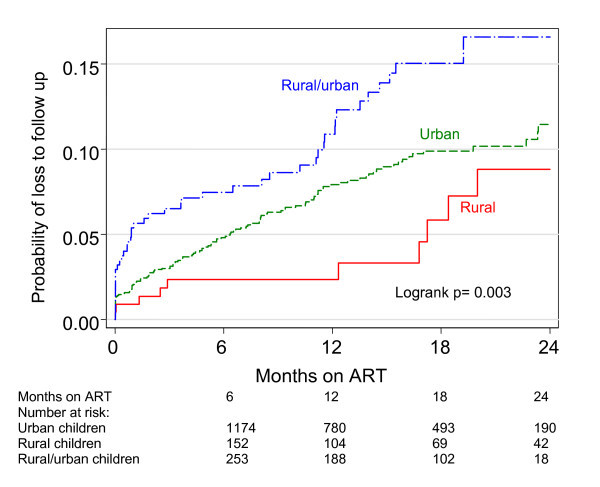
**Kaplan-Meier estimates of cumulative loss to follow up in rural and urban children**.

Overall 179 (7.7%) children became lost to follow up, 125 (7.2%) in the urban group, 10 (4.4%) in the rural group and 44 (11.7%) in the rural/urban group (χ^2 ^p = 0.002). After 24 months of ART, the probability of LTFU was almost two-fold higher among rural children travelling to urban treatment sites compared with those accessing care in rural areas, being 11.5% (95% CI: 9.3-14.0%), 8.8% (95% CI: 4.5-16.9%) and 16.6% (95% CI: 12.4-22.6%) in the urban, rural and urban/rural groups, respectively (logrank p = 0.0028) (Figure 2). For all groups, the instantaneous hazard of death was highest during the first six months of ART and levelled off over time on ART, while the instantaneous hazard of LTFU remained more constant over time.

Table 2 shows the results of univariate and adjusted regression models of baseline factors associated with death and LTFU over 24 months of ART. Forty-one (1.7%) children were excluded from the final adjusted models due to missing baseline tuberculosis treatment information. Children in the rural group had an independently increased probability of mortality, aHR 2.41 (95% CI: 1.25-4.67; p = 0.009) in comparison with urban children, after adjusting for baseline demographic and clinical variables. Severe clinical status, unavailable clinical status, severe immunodeficiency and missing baseline CD4 cell result were independently associated with increased probabilities of mortality. Children younger than 12 months had an increased probability of mortality compared with children older than two years.

**Table 2:** Factors associated with death and loss to follow up (LTFU) after 24 months of ART (n = 2291)a,b

Patient factor	HR (95% CI) of death		HR (95% CI) of LTFU	
	**Univariate**	**Adjusted**	**Univariate**	**Adjusted**

**Male gender**	0.86 (0.54-1.38)	0.83 (0.51-1.34)	1.06 (0.79-1.42)	1.1 (0.82-3.12)

**Age**				

>2 years	1	1	1	1

1-2 years	1.75 (0.89-3.46)	1.50 (0.74-2.98)	1.69 (1.11-2.57)	1.61 (0.96-2.68)

<1 year	3.72 (1.88-7.35)	2.73 (1.32-5.53)	1.87 (1.06-3.31)	1.81 (0.94-3.64)

**Severe clinical status**				

No	1	1	1	1

Yes	5.34 (3.07-9.43)	4.50 (2.52-8.00)	1.74 (1.23-2.46)	1.47 (1.03-2.12)

Not available	5.10 (2.62-9.93)	5.03 (2.37-10.68)	2.37 (1.62-3.50)	1.63 (1.02-2.59)

**Immunodeficiency**				

Not severe	1	1	1	1

Severe	8.71 (2.11-35.8)	6.77 (1.63-28.1)	0.85 (0.57-1.29)	0.81 (0.52-1.24)

Not available	8.80 (2.06-37.7)	4.74 (1.08-20.7)	1.68 (1.10-2.56)	1.18 (0.74-1.87)

**Tuberculosis at ART start**				

No	1	1	1	1

Yes	2.88 (1.24-6.67)	2.71 (1.15-6.34)	1.49 (0.73-3.02)	1.41 (0.69-2.86)

**Initial ART regimen**				

Non PI-based	1	1	1	1

PI-based	1.37 (0.80-2.37)	1.45 (0.72-2.96)	1.42 (1.02-1.98)	1.34 (0.88-2.04)

				

Non d4t-based	1	1	1	1

d4T-based	0.91 (0.33-2.52)	0.87 (0.32-2.54)	0.80 (0.46-1.42)	0.79 (0.44-1.44)

**Urban/rural classification**				

Urban	1	1	1.67 (0.89-3.19)	1.14 (0.57-2.24)

Rural	2.39 (1.31-4.36)	2.41 (1.25-4.67)	1	1

Rural/urban	1.17 (0.60-2.26)	1.02 (0.51-2.10)	2.38 (1.36-5.39)	2.85 (1.41-5.79)

^a^Adjusted hazard ratios are adjusted for all variables displayed in the table

^b^41 (1.7%) of children were excluded due to missing baseline tuberculosis treatment information

Rural children travelling to urban facilities (rural/urban group) had an independently increased probability of LTFU, HR 2.85 (CI: 1.41-5.79; p = 0.004) compared with children accessing care in rural areas, after adjusting for baseline variables.

The subgroup analyses selecting only children with all baseline variables available and using WHO stage and WAZ scores, instead of severe clinical status, included 1197 patients. There were no statistically significant differences in baseline demographic or clinical variables between this subgroup and the whole cohort. The probability of mortality in the rural group remained independently increased, aHR 4.23 (95% CI: 1.36-13.2; p = 0.013) within this subgroup. Children with WAZ scores <-3 had an independently increased probability of mortality, aHR 4.93 (95% CI: 1.84-13.2; p = 0.001; univariate HR 7.40 (95% CI: 3.12-17.6; p < 0.0001) in comparison with children with WAZ scores >-3. Children with WAZ scores between -3 and -1 were found not to have an increased probability of mortality compared with children with WAZ scores >-1 (p = 0.561), and no deaths occurred among 231 children with WAZ scores >0. There was no interaction between the effects of WAZ scores and the three groups on mortality.

The probability of LTFU in the rural/urban group compared with the rural group remained independently elevated, aHR 3.56 (95% CI: 1.63-7.77; p = 0.001) in the subgroup. There was no association between WAZ scores and LTFU (p = 0.50).

Among children with available viral load results, virological suppression was good, being 80.8% (95% CI: 78.3-83.1%; n = 1036) and 83.1% (95% CI: 76.6-88.5%; n = 66) after six and 24 months of ART, respectively. The rural/urban group had a lower proportion of patients achieving virological suppression (74.6%; 95% CI: 68.4-80.1%; p = 0.038) at any time point up to 24 months after starting ART, compared with 80.4% (95% CI: 78.5-82.2) in the urban group and 79.6% (95% CI: 71.8%-86.0%) in the rural group. Using a multivariate generalized estimating equation population-averaged model of virological suppression up to 24 months of ART adjusted for age, gender, clinical and immunological status, year of starting ART and initial regimen, the rural/urban group was associated with a reduced probability of virological suppression compared with the urban group, (OR 0.67; 95% CI: 0.48-0.93; p = 0.017, n = 1272 children) (Table 3). Children younger than two years had an independently reduced probability of virological suppression compared with children older than two years. Duration of time on ART was not associated with virological suppression (p = 0.20).

**Table 3:** Generalized estimating-equation model of baseline factors associated with virological suppression until 24 months of ARTa

Patient factor	Univariate odds ratio (95% CI)	Adjusted odds ratio (95% CI) (*n *= 1272)
**Male gender**	0.96 (0.78-1.19)	0.96 (0.77-1.19)

**Age**		

>2 years	1	1

1-2 years	0.48 (0.35-0.66)	0.53 (0.37-0.77)

<1 year	0.28 (0.18-0.43)	0.35 (0.21-0.56)

**Severe clinical status**		

No	1	1

Yes	0.59 (0.46-0.76)	0.65 (0.50-0.85)

Not available	0.60 (0.39-0.92)	0.56 (0.35-0.91)

**Immunodeficiency**		

Not severe	1	1

Severe	0.73 (0.56-0.96)	0.80 (0.60-1.01)

Not available	0.76 (0.54-1.07)	0.96 (0.65-1.41)

**Urban/rural classification**		

Urban	1	1

Rural	0.95 (0.62-1.46)	0.87 (0.55-1.35)

Rural/urban	0.71 (0.52-0.98)	0.67 (0.48-0.93)

^a^Adjusted odds ratios are adjusted for all variables displayed, as well year of starting ART, initial regimen and duration of time on ART of 6-24 months

After 12 months of ART, the median CD4 percentage increase was 12% (IQR: 7-17.6%) with no differences between the three groups (p = 0.88). The proportion of children with severe immunodeficiency at baseline was, however, higher in the rural group (p = 0.043), and remained higher up until the 12-month CD4 cell measurement (p = 0.20). (Six-month measurements were 41.8%, 57.7% and 36.5% in the urban, rural and rural/urban groups, respectively, p = 0.027.)

**Figure 3:**
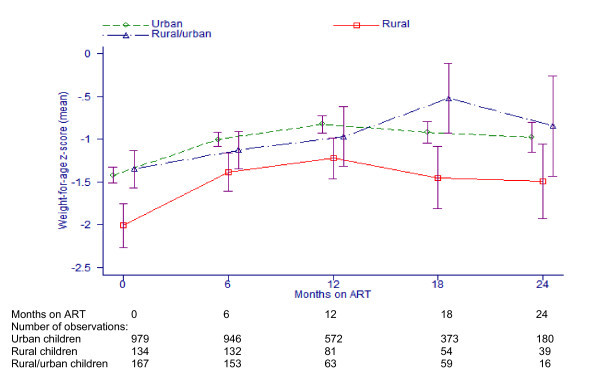
**Changes in mean weight-for-age z-scores after initiating ART in rural and urban children^a,b^**. ^a^Limited to children with measurement at ART start and at least one subsequent measurement. ^b^Error bars are 95% confidence intervals.

Children accessing care in rural areas had significantly lower baseline WAZ scores than children in other groups, and as illustrated in Figure 3 this difference persisted during treatment. Multivariate analysis of WAZ scores up to 24 months of ART, adjusting for baseline demographic and clinical variables, revealed that children in the rural group had independently reduced on-treatment WAZ scores compared with children in the urban group (co-efficient -0.42; 95% CI: -0.17 to -0.67; p = 0.001, n = 1756 children), i.e., over 24 months of treatment, the adjusted mean difference in WAZ scores modelled between rural and urban children was 0.4 points (lower in rural children). There was no interaction between the groups and duration of time on ART (p = 0.31), i.e., the rate of WAZ score increases on ART were equivalent between groups.

The proportions of eligible patients with available results of scheduled six-monthly on-treatment laboratory tests was significantly lower at rural health facilities than at urban facilities. (Viral load result availability was 23%-46% and 58%-75% at rural and urban facilities, respectively; CD4 cell result availability was 54%-72% and 71%-84% at rural and urban facilities, respectively).

## Discussion

This study has directly compared ART outcomes in rural and urban children in South Africa, and indicates that children in rural areas have an increased risk of poor outcomes on ART. Children receiving ART at rural facilities exhibited delayed access to ART, evidenced by their older age, more advanced immunosuppression, and greater degree of wasting when starting treatment than their urban counterparts. Rural Zambian children in contrast were younger and had less advanced immune suppression when starting treatment, although they also displayed higher levels of malnutrition than urban Zambian children [16].

### Mortality in rural children

In data from low-income settings globally, reported child mortality rates (including non-HIV-related mortality) are roughly one-third higher in rural children than in urban children, being influenced by reduced education, reduced access to safe water and sanitation, reduced vaccine coverage and a lack of healthcare services [25]. Rural childhood mortality in this dataset was, however, more than two-fold that of urban children, being similar to results from Zambia [16].

The reason for this is probably multi-factorial. First, mortality is strongly associated with baseline CD4 cell percentage [26]. Rural children had a reduced baseline CD4 cell percentage, and although median CD4 cell increases were equivalent between groups, a higher proportion of children at rural facilities had severe immunosuppression between months 0 and 6 of treatment, the period during which mortality rates were also the highest.

Second, poor nutrition is an independent risk factor for mortality in HIV-infected children [27,28] and is common in high HIV-prevalence rural areas of South Africa [29]. Adult ART programme mortality has also been reported to be higher in a rural South African setting than in urban settings, and was associated with low baseline weight [30]. The baseline nutritional status of rural children in this study was more impaired than that of urban children and differences persisted during treatment, which likely contributed to the higher mortality. Rural poverty, decreased access to high-protein food and greater environmental exposure to parasites are factors that reduce rural nutritional status.

Rural mortality remained elevated despite adjusting for available baseline clinical, nutritional and immunological variables, suggesting that other unmeasured factors were contributory causes. Anaemia is an independent risk factor for mortality in HIV-infected children [27], and has been found to be significantly more prevalent in rural than in urban HIV-infected Indian children [31]. Serum haemoglobin was not recorded in the routine monitoring data used in this study; however, poor nutritional status suggests that anaemia may further contribute to increased mortality in rural children.

Site-based facility factors were not directly measured in the study; however, the proportions of available clinical and laboratory values may be used as a proxy for site-based quality of care [32]. There were increased levels of unavailable baseline clinical and on-treatment CD4 cell and viral load results at rural facilities. Unavailable baseline clinical and immunological values were also independently associated with increased risks of mortality. Further research of the quality of paediatric ART care at rural facilities should therefore be conducted to assess if it is equivalent to care at urban facilities and to identify limitations.

### Loss to follow up in rural children attending urban facilities

LTFU was significantly increased in rural children managed at urban facilities. In Kwazulu-Natal Province, many rural ART facilities at both district hospital and primary healthcare level do not offer paediatric care, and children need to travel long distances to urban ART centres to access treatment. Long transport distances and times, lack of transportation, poor condition of roads, insufficient transport money [33] exacerbated by rural poverty [34] are barriers to the care of HIV-infected children in rural areas. Rural parents or carers on ART may attend a facility close to home, but need to take their children to another, distant urban-based facility, which is further likely to increase the risk of LTFU. Children in the rural/urban group are also less likely to have home-based adherence counsellor visits, as counsellors are networked with the facilities and do not support people long distances away.

Poor virological response is an important factor associated with disease progression and death, predisposes to the development of drug-resistance, and reduces future treatment options [35]. Rural children travelling to urban treatment centres had an approximately 30% decreased probability of virological suppression compared with urban children. This is likely due to lower ART adherence as a result of increased distances to health facilities, decreased frequency of visits and decreased or no contact with home adherence counsellors. In Limpopo Province, South Africa, rural children with longer travel distances and decreased adherence (self-reported and from pill counts) also demonstrated a trend towards virologic failure [11].

Overall programme outcomes, however, compare very favourably with results from other large cohorts in low-income settings [8,23,26,36], and the results reinforce that good childhood ART outcomes on a broad scale are possible in sub-Saharan Africa.

### Improving clinical outcomes and increasing uptake of ART

Paediatric treatment is still provided mainly at hospitals due to the availability of paediatricians and related support and a shortage of clinicians at the primary healthcare level. Lack of pharmacy staff at decentralized facilities is also a barrier, particularly for infants, as solutions currently need to be dispensed by a qualified pharmacist. Paediatric ART care can, however, be as effective at the primary healthcare level as hospital-based care, as has been demonstrated in the Western Cape in South Africa [37].

This data supports the process of shifting paediatric HIV care to the primary healthcare level, thereby increasing the number of service points and improving access and uptake of treatment, particularly in rural areas. To achieve this, there needs to be a focus on upskilling nurses and medical officers to initiate and maintain children on treatment through training and mentoring, improving access to laboratories, expanding community support, subsidizing transport for complicated cases, and establishing clear referral pathways with outreach specialist support. Furthermore, ensuring that all neonates exposed to HIV have HIV DNA polymerase chain reaction tests at their first vaccine visits at six weeks, and are followed up at 10 weeks with urgent referral to an ART facility if found to be positive will likely lead to decreased early childhood mortality [38].

### Strengths and limitations

The strengths of this study are that pooled data from a large number of patients and sites in different settings were used, and individual-level data was collected prospectively enabling exploration and adjustment of patient factors associated with outcomes. This study is a retrospective analysis using routine data with its inherent limitations; however, it is likely to be indicative of the situation at an operational level. Missing viral load results were prevalent, particularly at rural sites, which may bias viral suppression estimates; the proportion of available results was, however, higher than those from a number of other sub-Saharan ART programmes [39]. All children were eligible for laboratory testing at six-monthly intervals, irrespective of clinical condition in accordance with South African national guidelines, and all available results were due to be captured in the database. Therefore, any bias due to missing laboratory results is expected to be non-systematic.

The sites included were all supported by an NGO, and it is possible that the outcomes may not be easily generalizable to non-NGO-supported government health facilities. Adherence determination data (such as pill counts) was not collected as they do not form part of the routine data captured for public-sector ART patients in South Africa. A proxy measure of adherence, such as pharmacy refill data, was not included in this study. It is possible that ascertainment of death was more complete at rural (smaller) than urban (larger) sites. However, as the vital status of patients LTFU in this study was not traced, this could not be accurately determined. In addition, data on distance travelled to clinics and relocations were not available. Socio-economic factors may additionally be associated with mortality, but it was not possible to collect socio-economic data for the whole cohort for this study and it was not possible to include this variable in analyses.

## Conclusions

Rural HIV-infected children are a vulnerable group, and providing care in rural areas poses significant challenges. Most studies investigating the outcomes of ART programmes involve urban children, and almost three-quarters of children in this study lived in urban areas. Future research of rural paediatric ART programmes is important and should examine the mechanisms underpinning the observed vulnerability, explore cost-effective improvements and efficiency in health systems, and determine the degree to which HIV-infected children and their families in rural settings require additional nutritional, healthcare and social support.

## Competing interests

The authors declare that they have no competing interests.

## Authors' contributions

GF, PB and BE designed the study. GF analyzed the data. All authors interpreted the data. All authors contributed to the writing of the manuscript, and all authors approved the manuscript for publication.
